# Two new species of *Elatostema* (Urticaceae) from southeast Yunnan, China

**DOI:** 10.3897/phytokeys.7.2022

**Published:** 2011-11-29

**Authors:** Zeng-Yuan Wu, Wen-Tsai Wang, Hong Wang, De-Zhu Li

**Affiliations:** 1 Key Laboratory of Biodiversity and Biogeography, Kunming Institute of Botany, Chinese Academy of Sciences, Kunming Yunnan 650201, China; 2 State Key Laboratory of Systematic and Evolutionary Botany, Institute of Botany, Chinese Academy of Sciences, Beijing 100093, China; 3 Germplasm Bank of Wild Species, Kunming Institute of Botany, Chinese Academy of Sciences, Kunming, Yunnan 650201, China; 4 Graduate University of the Chinese Academy of Sciences, Beijing 100049, China

**Keywords:** China, *Elatostema*, new species, Urticaceae, Yunnan Province

## Abstract

*Elatostema pleiophlebium* and *Elatostema malipoense,* two endemic species from southeast Yunnan of China, are described and illustrated. Their diagnostic characters, description and relationship with morphologically similar species are also given. *Elatostema pleiophlebium* is easily recognised by its glabrous stem and leaf blade, and its longitudinally 1-ribbed outer staminate bracts. *Elatostema malipoense* is morphologically distinct for its tuberculate achene and pistillate receptacle which is puberulous. Both new species are known only from their type localities, and they are proposed to be classified as critically endangered.

## Introduction

The genus *Elatostema* J. R. Forst. & G. Forst. is one of the largest genera of the family Urticaceae. This genus is characterized by the perianth lobes of female flowers being much shorter than the ovary or strongly reduced, and not corniculate at apex (Chen et al. 2003; Wang 1995). The staminate inflorescences play an important role in the delimitation of sections, but for delimiting series leaf venation, peduncle length, achene morphology and leaf reduction of staminate stem are used as main distinguishing characters (Wang 2011a).

*Elatostema* comprises ca. 550 species distributed in tropical and subtropical Africa, Asia and Oceania, especially in humid areas (Chen et al. 2003, Wang 2011a). So far, 233 species (205 endemic) have been recorded for China. These mainly occur in the tropical and subtropical regions south of the Qinling Mountains (Wang 1995, 2011a). More than seventy species of *Elatostema* have been recorded for southeast Yunnan (e.g. Wang 1997, 2003, 2006, 2010, 2011b; Wu et al. 2011), which was defined as one of the plant endemism centres in China (López-Pujol et al. 2011). During botanical surveys conducted by the authors in this region, two hitherto undescribed species were encountered, which are described and illustrated here.

### Elatostema pleiophlebium

W.T.Wang & Zeng Y.Wu, sp. nov.

urn:lsid:ipni.org:names:77116005-1

http://species-id.net/wiki/Elatostema_pleiophlebium

Fig. 1F–I 2A–C 

#### Latin

Ob foliorum nervos laterales plures et capitula staminata 6-bracteata glabra species nova haec est fortasse affinis *Elatostema quinquecostato* W.T.Wang, quod caulibus strigosis, foliis apice cuspidatis supra hispidis subtus strigosis nervos laterales utrinsecus usque ad 12-14 ferentibus, capituli staminati bracteis duabus exteris dorso 5-costatis et infra apicem breviter corniculatis praeclare differt.

#### Type.

China. Yunnan, Hekou county, Nanxi village, Sanchahe river, 22°41'4"N, 103°59'26"E, 388 m, 01 Aug. 2010, Z. Y. Wu 10181 (Holotype: PE!; Isotype: PE!); the same locality, 01 Aug. 2010, Z. Y. Wu 10186 (Paratype: KUN!).

#### Description.

Perennial herb. Stems erect, 30-50 cm tall, glabrous, unbranched. Stipules narrowly lanceolate or lanceolate, 5–14 × 1.5–3 mm, with cystoliths 0.5–0.7 mm long, apex pungent; leaves shortly petiolate, glabrous, petioles 3–17 mm long; leaf blade chartaceous, obliquely narrowly ovate, broadly oblong or elliptic, 10–20 × 4.5–7.2 cm, glabrous, adaxial surface with 1 broad, interrupted, white stripe along the mid vein, adaxial surface with dense cystoliths, conspicuous, bacilliform, 0.25–0.8 mm long, penninerved, narrow side with (4-) 7–10 lateral nerves, broad side with (5-) 7–11 lateral nerves, base obliquely cuneate, margin denticulate, apex acuminate, shortly acuminate or obtuse. Staminate capitula solitarily axillary, glabrous; peduncle ca. 1.5 mm long; receptacle broadly oblong, ca. 8 × 6 mm; bracts 6, 2-seriate, ovate or narrowly ovate, abaxially above longitudinally 1-ribbed, with rib apex extended into subulate horn-like projections, outer 2 opposite, larger, 4–5 × 9 mm, with apex projection 3 mm long, inner 4 smaller, 4–5 × 5–7 mm, apex projection 1–1.5 mm long; bracteoles membranous, numerous, semihyaline, above brownish, obtrapezoid or navicular, 2–3.2 × 0.6–2 mm, above slightly conduplicate, apex cucullate. Staminate flower buds subsessile, broadly obovoid, ca. 2 mm long, glabrous, apex 4-corniculate. Female flowers and achenes not known.

#### Ecology.

*Elatostema pleiophlebium* is a forest understory herb. In the type locality, it occurs on wet ground in forest close to a river where it is associated with *Elatostema alnifolium* and some species of *Musa*, *Ficus* andPiperaceae.

#### Distribution and conservation status.

*Elatostema pleiophlebium* is known only from the type locality near the Sanchahe river, Nanxi village, Hekou county, Yunnan. Consequently, it is probably an endemic species. A single population of a few hundred individuals was observed in an area of 1 km^2^. We believe therefore, that this new species is on the verge of extinction but we do not know if population size is stable or declining. Following the IUCN red list criteria (IUCN 2011), we propose to classify this species as critically endangered (CR B2ab (iii); C2b).

#### Similar species.

*Elatostema pleiophlebium* is a member of series Nanchuanensia W.T.Wang in sect. *Elatostema* (Wang 2011a). *Elatostema pleiophlebium* is similar to *Elatostema quinquecostatum* W.T.Wang in having numerous lateral leaf nerves and a staminate capitulum involucre formed of six glabrous bracts. *Elatostema quinquecostatum* differs from *Elatostema pleiophlebium* in having stems with strigose hairs, cuspidate leaf apices, hirsute adaxial leaf surface, strigose abaxial leaf surfaces, bearing ca. 12–14 nerves, the outer 2 staminate bracts abaxially longitudinally 5-ribbed and short coniculate below apex (Wang 1995).

#### Etymology.

The epithet ‘pleiophlebium' refers to the numerous lateral nerves characteristic of the leaves of this species.

**Figure 1.**
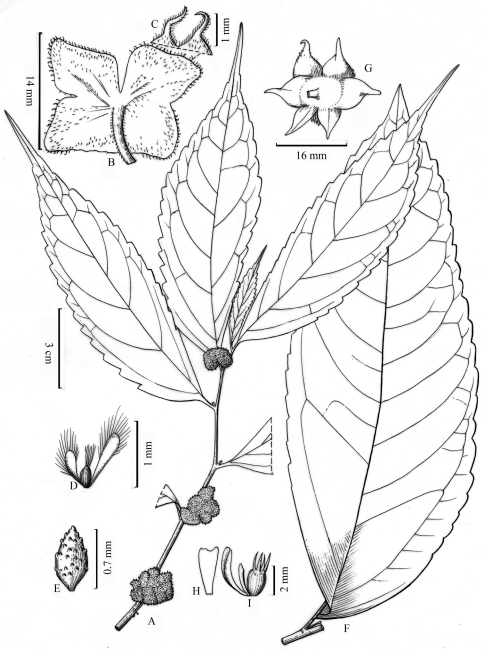
**A–E** *Elatostema malipoense*W. T. Wang & Zeng Y. Wu. **A** Upper part of flowering pistillate stem **B** pistillate capitulum, seen from below **C** two pistillate involucral bracts **D** pistillatate bracteoles and pistillate flower **E** achene, (based on Z. Y. Wu 10347). **F–I** *Elatostema pleiophlebium* W.T.Wang & Zeng Y.Wu. **F** upper cauline leaf **G** staminate capitulum **H** staminate bracteole **I** staminate bracteoles and staminate flower bud, (based on Z. Y. Wu 10181).

### Elatostema malipoense

W.T.Wang & Zeng Y.Wu, sp. nov.

urn:lsid:ipni.org:names:77116006-1

http://species-id.net/wiki/Elatostema_malipoense

Figs 1A–E 2D–G 

#### Latin

Ob folia penninervia et capituli pistillati bracteas numerosas apice corniculatas species nova haec est similis *Elatostema pseudobrachyodonto* W.T.Wang, quod foliis supra puberulis, capituli pistillati receptaculo glabro, bractearum pistillatarum cornibus apicalibus majoribus 1.5-2 mm longis, bracteolis pistillatis viridibus minoribus 0.3–0.6 mm longis apice breviter ciliatis, acheniis longitudinaliter 4-costatis valde recedit.

#### Type.

China. Yunnan, Malipo county, Xiajinchang village Yunling, 23°10'6"N, 104°49'50"E, 1613 m, 05 Aug. 2010, Z. Y. Wu 10347 (Holotype: PE!; Isotype: KUN!).

#### Description.

Perennial herb. Stems erect, 30-50 cm tall, above sparsely short- puberulous near the node, unbranched. Stipules subulate or narrowly triangular, 0.1–0.2 mm long; leaves shortly petiolate, glabrous, petioles 1–6 mm long; leaf blade subchartaceous, obliquely oblong or narrowly obovate-oblong, 11–15 × 3–3.5 cm, both surfaces densely short strigose, cystoliths conspicuous, dense, bacilliform, 0.1–0.2 mm long, penninerved, lateral nerves 5–7-paired, base obliquely cuneate, apex cuspidate (entire), margin denticulate. Pistillate capitula solitarily axillary; peduncle ca. 8 mm long, short-puberulous; receptacle subquadrate or broadly oblong, 10–15 × 10–15 mm, 4-lobulate or irregularly 4–6-lobulate, short-puberulous; bracts ca. 75, deltoid or depressed-deltoid, 0.5–0.6 × 0.5–0.7 mm, ciliate, abaxial surface short-puberulous, apex with a subulate horn-like projections, 0.7–1 mm long; bracteoles membranous, whitish, semihyaline, narrowly obovate or oblanceolate, 0.5–1 mm long, apically long ciliate. Pistillate flower subsessile, tepals absent; pistil 0.8 mm long, ovary green, 3.5 mm long, stigma penicillate, ca. 4.5 mm long. Achenes ovoid, 0.6–0.7 × 0.4 mm, densely tuberculate. Staminate capitula not seen.

#### Ecology.

*Elatostema malipoense* is a scattered understory herb growing in moist clay soils in shady sites or near ravines at an altitude of ca. 1600 m, associated with *Pilea insolens* and some species of *Ficus*.

#### Distribution and conversation status.

*Elatostema malipoense* is an endemic species and has only been collected from the type locality around Xiajinchang village Yunling, Malipo county, Yunnan, where a population of ca. 200 individuals was observed in an area of 1 km^2^. According to IUCN red list criteria (IUCN 2011), this new species should be classified as critically endangered (CR B2ab (iii); C2b).

#### Similar species.

*Elatostema malipoense* is a member of sect. *Elatostema* (Wang 2011a). In having penninerved leaves and a pistillate capitulum with numerous corniculate involucral bracts, *Elatostema malipoense* resembles *Elatostema pseudobrachyodontum* W.T.Wang. *Elatostema pseudobrachyodontum* differs from *Elatostema malipoense* in having short-puberulous adaxial surface of the leaf blade, glabrous pistillate receptacles, pistillate bracts 1.5-2 mm long, and corniculate, pistillate bracteoles are green, 0.3-0.6 mm long, apex short-ciliate at the apex and achenes that are longitudinally 4-ribbed (Wang 1995).

#### Etymology.

The species epithet ‘malipoense' is derived from the name of the type locality, Malipo County, Yunnan Province, China.

**Figure 2.**
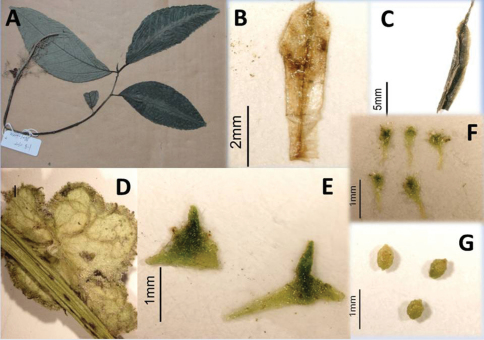
**A–C** *Elatostema pleiophlebium* W.T.Wang & Zeng Y.Wu. **A** Specimen **B** staminate bracteole **C** stipule **D–G** *Elatostema malipoense* W.T.Wang & Zeng Y.Wu. **D** pistillate capitulum, seen from below **E** pistillate involucral bracts **F** pistillate bracteoles **G** achenes.

## Supplementary Material

XML Treatment for Elatostema pleiophlebium

XML Treatment for Elatostema malipoense
